# The Chinese version of the Computer Vision Syndrome Questionnaire: translation and cross-cultural adaptation

**DOI:** 10.1186/s12886-023-03031-y

**Published:** 2023-07-03

**Authors:** Natalia Cantó-Sancho, Mar Seguí-Crespo, Guanlan Zhao, Elena Ronda-Pérez

**Affiliations:** 1 Department of Optics, Pharmacology and Anatomy, University of Alicante, San Vicente del Raspeig, 03690 Spain; 2 Public Health Research Group, University of Alicante, San Vicente del Raspeig, 03690 Spain; 3 Department of Social Medicine and Health Management, School of Public Health, Lanzhou University, Lanzhou, 730000 China; 4 Biomedical Research Networking Center for Epidemiology and Public Health (CIBERESP), Madrid, 28029 Spain

**Keywords:** Cross-cultural adaptation, Computer vision syndrome, Questionnaire, Video display terminals, Public health, Chinese

## Abstract

**Background:**

The Spanish version of the Computer Vision Syndrome Questionnaire (CVS-Q^©^) is a validated instrument, with good psychometric properties, to measure Computer Vision Syndrome (CVS) in workers using Video Display Terminals (VDTs). To date, there are no known valid instruments in Chinese for the assessment of CVS despite the high exposure to VDTs at work that this population presents. For this, the purpose of this study is to translate and cross-culturally adapt the CVS-Q^©^ into Chinese.

**Methods:**

A study with five consecutive stages: direct translation, synthesis of translations, back translation, consolidation by a committee of experts, and pre-test. During the pre-test, a cross-sectional pilot study was conducted on VDT users (*n* = 44) who completed the Chinese version of the questionnaire plus an *ad hoc* post-test to assess the comprehensibility of the scale and to verify aspects of its applicability and feasibility. Data concerning sociodemographic information, general and ocular health, use of optical correction and varying exposure to VDTs was also collected.

**Results:**

The entire sample considered the Chinese version of the CVS-Q^©^ simple, clear, and easy to understand and 95.5% also found it easy to complete. 88.7% considered that the scale did not need any improvement. The final version of the Chinese scale to measure CVS was obtained (the CVS-Q CN^©^). The mean age of participants was 31.3 ± 9.8 years, 47.6% were women, and 57.1% used VDTs to work for more than 8 h/day.

**Conclusions:**

The CVS-Q CN^©^ can be considered an easy tool to assess CVS in workers exposed to digital devices in China. This version would facilitate research, its use in clinical practice, and the prevention of occupational hazards in the workplace.

**Supplementary Information:**

The online version contains supplementary material available at 10.1186/s12886-023-03031-y.

## Background

With the rapid development of the information era, more and more digital devices are gradually becoming part of people’s lives. There is no doubt that informatization has brought convenience to our daily lives, but at the same time, it can also contribute to eye problems. The computer screen is composed of many small light spots, and it is not easy to focus the eyes in front of a screen that flickers frequently, so the appearance of ocular and visual symptoms is very common. There is a risk that Computer Vision Syndrome (CVS) will become a major public health issue around the world [1]. According to the definition of the American Optometric Association, CVS, also known as digital eye strain, is defined as a complex of eye and vision problems related to the prolonged use of digital devices [2].

China, commonly known as the world’s second largest economy, is home to almost a quarter of the world’s population. The China Internet Development Statistical Report showed that the number of netizens has reached 1.01 billion, and the population of 30–39 occupied the highest proportion of all age groups with a rate of 20.3%. The proportion of citizens aged 40–49 and 20–29 is 18.7% and 17.4%, respectively, and between all age groups, 95.2% of enterprises use computers in their daily work in China [3]. According to the Chinese Occupational Health Examination Regulation, the working population using Video Display Terminals (VDTs) have been included in occupational health monitoring.

In a global context, there are still many discussions about CVS measurement tools. The main limitation of the studies published so far is that they evaluate CVS through invalidated and unstructured questionnaires, which include different symptoms according to the authors and imprecise and different definitions of when a worker should be considered symptomatic [4], enormously compromising the findings.

Concretely in China, there are different studies that have examined how prolonged use of VDTs influences the workers’ health [3, 5, 6], and, specifically their impact on ocular and visual health [7, 8]. There are even clinical trials looking for treatment that would reduce CVS [9] or guidelines on health food exportation to China, supporting healthy foods to alleviate CVS. However, among Chinese workers, the main studies have examined individual ocular and visual symptoms, without considering CVS as a global issue or without using validated or specific instruments for the diagnosis of CVS, which compromises the reliability of its results. For example, we found the Cheng et al. [3] study that concludes that when the daily operation time of the VDT was more than 11 h, Chinese internet staff were more likely to suffer from dry eye (OR = 2.22, 95%CI = 1.17–4.20) and ocular soreness (OR = 2.16, 95%CI = 1.01–4.61). Another study was the research by Yu et al. [7] study in which they used a questionnaire to evaluate visual fatigue developed by Kuze et al. [10], which includes 5 subscales, which, not only assesses eye strain, but also includes symptoms of general discomfort, nausea, difficulty in focusing, and headaches. Moreover, in China, the CVS study has focused more on the student population and these studies do not use standardized questionnaires either, so the results may not be valid or generalizable [11–13]. Recently, some Asian authors have focused on using a completed tool to measure CVS as a global construct, the Computer Vision Syndrome Questionnaire (CVS-Q^©^) [14–16]. However, the CVS-Q^©^ scale they used was originally designed in Spanish and, although it has good psychometric properties to diagnose CVS [17], it has not been translated and culturally adapted into Mandarin Chinese. Furthermore, in the study by Toh et al. [16] the original CVS-Q^©^ has been modified without apparent criteria, including a different set of symptoms according to the author.

Due to the abovementioned problems, the aim of this study is to translate and cross-culturally adapt the CVS-Q^©^ into Chinese to provide both researchers and healthcare professionals with a tool for assessing CVS.

## Methods

### Computer Vision Syndrome Questionnaire (CVS-Q^©^)

In 2015, Seguí et al. developed and validated a self-administered Spanish questionnaire to evaluate CVS, the CVS-Q^©^ [17]. The questionnaire was drawn up based on a review of the scientific literature and was developed and validated with the broad consensus and acceptance of experts in different fields (clinic medicine, epidemiology, optometry and ophthalmology). The pre-test, a pilot test, and a new evaluation were also completed. The CVS-Q^©^ has good psychometric properties derived from Rasch’s analysis, with sensitivity and specificity values of 75.0% and 70.2%, respectively. Therefore, for these reasons, it can be considered a valid and reliable tool to assess CVS in workers exposed to VDTs. This questionnaire assesses the frequency and intensity of 16 ocular and visual symptoms related to the use of VDTs. The frequency and intensity of use data are recorded to calculate the severity of each symptom, resulting in a total score. Total scores ≥ 6 indicate that the subject has CVS.

### Stages of the study

**Fig. 1:**
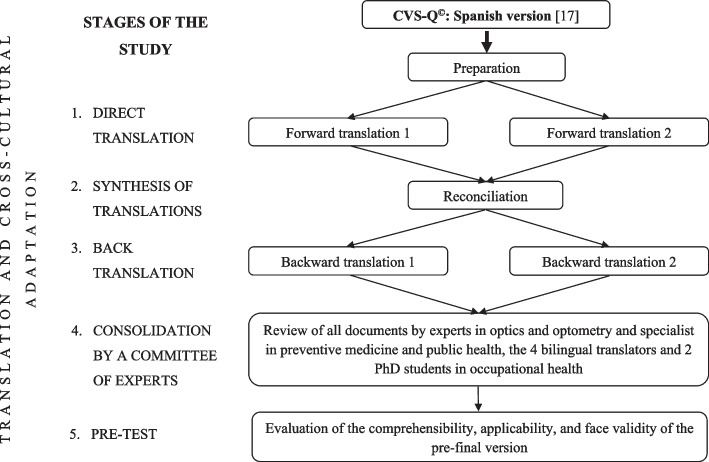
Flowchart of the translation and cross-cultural adaptation stages of CVS-Q^©^ into Chinese

This study consists of 5 differentiated, complementary, and sequential stages, which follow the methodology recommended by the scientific literature [18, 19] and the same process as we have followed for other translations and cultural adaptations of the CVS-Q^©^ into different languages [20, 21]. The stages carried out are as follows (Fig. 1):

1. Direct translation. A full translation of the original questionnaire into the target language was carried out independently by two bilingual (mother tongue Chinese) translators.
2. Synthesis of translations. The two translators who had participated in the previous stage (T1 and T2) met online to synthesize and compare both translations, identify the terms or expressions that varied between the translated versions and reach a consensus to obtain a single version of the questionnaire in Chinese.
3. Back-translation. The synthesis version obtained in the previous stage was back-translated into Spanish (original language). It was carried out by two bilingual, native Spanish translators who had not participated in the two previous stages (RT1 and RT2). Both translators worked independently and without reference to the original CVS-Q^©^ (blinding).
4. Consolidation by a committee of experts. A multidisciplinary committee was formed consisting of an expert in optics and optometry and a specialist in preventive medicine and public health (both authors of the original questionnaire), the 4 bilingual translators (T1, T2, RT1, and RT2) who had participated in the previous stages, two PhD students in occupational health, who have been working for several years in the field of CVS and a doctor in both the field of general practice and the field of public health. All translations (direct and back), the synthesis version, and the original questionnaire were made available to the committee. The role of the committee was to identify the discrepancies found, reach a consensus, obtain a single consolidated pre-final questionnaire which has been culturally adapted to Chinese and is conceptually equivalent to the original questionnaire and will present adequate content validity.
  In different reports, all the information from the 4 stages carried out was compiled and the reasons for each decision were recorded.
5. Pre-test (applicability/feasibility). The pre-final version of the questionnaire obtained in the previous stage was subjected to a preliminary test or pre-test, to evaluate the quality of the translation and cultural adaptation, check its comprehensibility, verify aspects of its applicability or feasibility, and evaluate its face validity.

A cross-sectional pilot study was conducted in a convenience sample of 44 participants (42 adults and 2 adolescents) from different regions of China, who were contacted during the months of June to September 2021. Inclusion criteria were participants of both sexes, aged 18–65 years, with different occupations/educational levels, who spoke Chinese as their first language, and who could read and understand what they read, all of whom were exposed to VDTs at some point during their day.

The purpose of the study was explained to all people. The included participants gave their explicit written consent to participate. The study was approved by the University of Alicante (UA-2018-02-22) and was conducted following the standards of good clinical practice and international ethical principles applicable to human research, according to the latest revision of the Declaration of Helsinki. In addition, the Organic Law 3/2018, of 5th December, on Personal Data Protection and the guarantee of digital rights were both considered.

All participants completed an online anamnesis questionnaire that collected sociodemographic information (sex, age, educational level and occupation), general and ocular health (systemic and ocular pathologies, general and ocular pharmacological treatments and ocular surgeries), use of optical correction (use of glasses and/or contact lenses regularly and at work), use a VDT (h/day at work using a VDT, scheduled breaks while using a VDT and h/day using a VDT for leisure activities), as well as an adapted online version of the pre-final CVS-Q^©^ in Chinese and an *ad hoc* designed post-test composed of closed and open questions, where participants were invited to comment on any aspects of the Chinese version of the CVS-Q^©^ that they found difficult to understand. Additionally, each participant was asked to measure the time it took to complete the questionnaire and indicate this. All possible difficulties in understanding the questionnaire instructions, symptoms, and response options were identified, and finally a report was prepared by grouping common discourses. From there, all parts of the questionnaire where at least 15% of the participants expressed difficulties or suggested changes were revised.

For statistical analysis, a descriptive analysis of categorical variables was performed by calculating the absolute frequency and percentage. For continuous variables, the mean, standard deviation (SD) and range were obtained. SPSS Statistics 28 statistical software was used for the analyses.

## Results

**Fig. 2:**
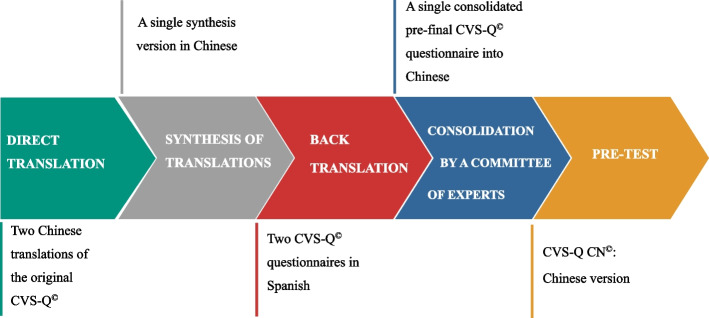
Main results of the different stages of translation and cultural adaptation of the CVS-Q^©^ into Chinese

The main results obtained in each of the different stages of translation and cultural adaptation are as follows: 1) from direct translation, two Chinese translations of the original CVS-Q^©^ questionnaire were obtained, as well as doubts about terms or phrases that arose, 2) as a result of the synthesis of translations, and after the translators reached a consensus, a single synthesis version in Chinese was obtained and a report indicating how they had resolved each of the discrepancies was produced, 3) the back-translation resulted in two CVS-Q^©^ questionnaires in Spanish, where the most difficult terms or concepts were indicated, 4) using all this information the expert committee reached a pre-final consolidated questionnaire adapted to Chinese with good content validity. This version was tested in the pre-test stage (Fig. 2).

The mean age of the adults who participated in the pre-test (*n* = 42) was 31.3 ± 9.8 years (range: 19–63 years) and 47.6% were female. Most of the participants had a bachelor’s degree, a master’s degree or a higher education degree (90.5%), and were faculty or corporate employees (61.9%). 28.6% of the sample had some systemic pathology, the most frequent being thyroid-related pathologies (*n* = 3) and allergies (*n* = 7). 23.8% reported some ocular alteration, mainly dry eye (*n* = 5) and amblyopia (*n* = 4), although the latter was in the past. None of the participants were taking any ocular pharmacological treatment. Concerning regular use of optical correction, those who indicated that they wore glasses occasionally or frequently (28.7%), the majority used them for reading, working and/or driving. Some 21.4% of the sample wore contact lenses occasionally (weekends and/or sport). 69.0% of the sample preferred to work wearing glasses rather than contact lenses. The mean VDT use for work or study was 8.9 ± 2.8 h/day (range: 4–15 h/day). Only 40.5% of the sample took scheduled breaks when using VDTs for work or study. For leisure purposes, the sample used VDTs an average of 2.7 ± 1.6 h/day (range: 1–8 h/day) (Table 1).

**Table 1:** Sociodemographic, general and ocular health, optical correction and video display terminal use characteristics of workers who participated in the pre-test (*n* = 42)

Variable	n	%
*Sex*		
Female	20	47.6
Male	22	52.4
*Age (years)*		
≤ 30	21	50.0
> 30	21	50.0
*Educational level*		
Up to bachelor level	4	9.5
With bachelor’s, master’s or higher education level	38	90.5
*Occupation*		
Faculty	14	35.7
Students	9	21.4
Corporate employees	11	26.2
Health workers	2	4.8
Others	5	11.9
*Systemic pathology*		
Yes	12	28.6
No	30	71.4
*General pharmacological treatments*		
Yes	3	7.1
No	39	92.9
*Ocular pathology*		
Yes	10	23.8
No	32	76.2
*Ocular surgery*		
Yes	3	7.1
No	39	92.9
*Use of regularly optical correction (glasses)*		
No	13	31.0
Occasionally	5	11.9
Frequently	7	16.7
Always	17	40.5
*Use of contact lenses*		
Yes	9	21.4
No	33	78.6
*Use of optical correction to work*		
No	12	28.6
Glasses	29	69.0
Contact lenses	1	2.4
*Use of VDTs to work or study (h/day)*		
≤ 8	18	42.9
> 8	24	57.1
*Schedules breaks while using VDTs*		
Yes	17	40.5
No	25	59.5
*Use of VDTs for leisure activities (h/day)*		
≤ 2	23	54.8
> 2	19	45.2

*VDTs*  Video display terminals

In addition, the two minors, a 10-year-old girl and an 11-year-old boy, used a VDT for 2 and 3 h/day, respectively.

The CVS-Q CN^©^ (Chinese version of the CVS-Q^©^) was rated by 100% of the participants as simple, clear, and easy to understand. Regarding whether it was easy to complete, 95.5% (*n* = 42) of the participants indicated that it was (including the minors). However, 2 people commented that they experienced some difficulty in completing it, one indicated that the questionnaire was too complicated in general and the other that the CVS-Q CN^©^ image was not visible enough (a problem derived from the fact that the administration was online). Almost the entire sample indicated that they had no problems understanding the technical terms of the instrument (*n* = 41). However, 3 participants indicated that they did (two adults and one minor). One adult did not understand symptoms 12 (difficulty focusing for near vision), 13 (increased sensitivity to light) and 14 (coloured halos around objects). The other adult did not understand item 10 (blurred vision). The minor did not understand items 1 (burning), 12 (difficulty focusing for near vision) and 14 (coloured halos around objects), but neither participant indicated any alternative terms to replaced them. In terms of possible improvements, 88.7% (*n* = 39) felt that the questionnaire did not need any improvement (including the minors). 5 participants commented that it did. Of these, 3 indicated that the questionnaire was made up of too many items/questions. One of them recommended reducing the number of items, another said that it would be advisable to add a short explanation behind each item so that people would not have doubts when answering a symptom-related question. Another commented that there should be more response options for both the frequency and intensity of symptoms. 42 people indicated that symptoms should not be added to the questionnaire (including the minors), while one person indicated that the symptom of “eye puffiness” should be added as it was noticed by the participant after prolonged use of VDTs. Another participant indicated that the symptom “general tiredness” should be added as he indicated, “when I use the computer for a long time, I yawn all the time, I want to sleep, and sometimes I feel very tired”. No participant commented that any symptom should be removed from the questionnaire. However, participants did not reach the 15% recommended by the literature to make any changes to the questionnaire. Therefore, the final version of the CVS-Q^©^ in Chinese, called: 电脑视觉综合征调查问卷 ; CVS-Q CN^©^ (see Additional file 1, also available in English), turned out to be the same as the pre-final version. The face and content validity has been demonstrated after carrying out the whole process. The mean time of completion was 4.03 ± 2.92 min (range: 1.30–12 min).

## Discussion

This study presents the translation and cultural adaptation process of the CVS-Q^©^ into Chinese for its use in the working population using VDTs, using widely used recommended guidelines in the scientific community. The face and content validity of the CVS-Q CN^©^ has been demonstrated by the comments of the experts, the original authors of the instrument, as well as by the considerations of the study population who rated the questionnaire as simple, clear, easy to understand and complete. Likewise, its applicability or feasibility has also been demonstrated.

For a questionnaire designed in another language to be used in a particular country (with a different language or culture), it must undergo a rigorous process of translation, cultural adaptation and validation in the target language [18]. The Chinese version of the CVS-Q^©^, having undergone a rigorous process with systematic steps to carry out translation and cultural adaptation, overcomes the limitations of the other instruments used to date in China to measure CVS: the use of non-CVS-specific questionnaires, the use of instruments that include a range of individual symptoms without considering CVS as a global construct, the modification of existing questionnaires to assess CVS with no apparent reason, and the use of questionnaires developed in another language without prior translation and cultural adaptation to guarantee their face and content validity.

To date, the original CVS-Q^©^ has been translated and cross-culturally adapted into different languages, such as English [17], Italian [21], Slovak [20] and Persian [22] following the guidelines developed by Beaton et al. [19] and the Translation and Cultural Adaptation group [23], which are also followed by other researchers worldwide [24–27]. In a recent systematic review, it has been observed that when a questionnaire is used in the Chinese population, and was originally designed in a non-Chinese language, one of the weaknesses is the lack of good cultural and language adaptation [28]. In our case, following a standardized process ensures that cultural aspects have been considered in each of the items to minimize errors due to misinterpretation of the items’ content. In addition, in the translation and cultural adaptation of this new version, an attempt has been made to eliminate the limitations made in the translation and cultural adaptation of the CVS-Q^©^ into other languages. Therefore, only bilingual translators have been contacted (which could not be done in the Persian version) [22]. A good team of bilingual translators is essential to reflect the nuances of the language more accurately in the target language and to be able to carry out an adequate translation and cultural adaptation. These translators had different profiles and came from different fields of knowledge; the direct translation involved a health specialist, who was proficient in the technical terms used and the conceptual framework related to CVS (his participation ensured a better cultural and linguistic equivalence between the original and the final questionnaire), and a specialist in Biomedicine, who provided a translation more in line with the colloquial language, which allowed difficulties encountered in the translation of more technical or uncommon terms to be resolved. The back-translation was done by a professional translator living in Spain, a Chinese teacher, and an intermediary between Spanish and Chinese companies who works as a manager at the Spanish Institute of Foreign Trade (ICEX), based in Hong Kong, a Chinese Special Administrative Region. Furthermore, two teenagers also participated in the study (which were not included in the Italian version) [21]. For a questionnaire to be truly comprehensible, it is indicated that it should be understood by individuals between 10 and 14 years of age, even if the questionnaire is not directed at this target population [29].

Regarding the limitations of this study, it should first be noted that not all participants in the expert committee were bilingual, as recommended in the literature, but those who participated in the translation were bilingual. In addition, almost the entire sample indicated that the questionnaire was easy, perhaps because the educational level of almost all participants was high. This could lead to the questionnaire not being easy for workers using VDTs with lower levels of education. However, this was addressed by the participation of two minors. Furthermore, one of the difficulties we found in this research is that the questionnaire could not be presented in a paper format, but an online adaptation was needed due to the pandemic. This caused some participants to comment on some of the difficulties in the pre-test, e.g., the image of the questionnaire was not clear.

## Conclusions

The CVS-Q CN^©^ can be considered as an easy-to-understand and simple-to-use tool to measure CVS in the working population who use VDTs in China. Although its face and content validity are assured, this process needs to be extended with a larger sample study to further demonstrate the adequacy of its psychometric properties.

## Supplementary Information

**Additional file 1. **The CVS-Q CN^©^; 电脑视觉综合征调查问卷^© ^– The Computer Vision Syndrome Questionnaire in Chinese (and its English version). It is a self-administered questionnaire in Chinese to evaluate the computer vision syndrome (or digital eye strain). This questionnaire assesses the frequency and intensity of 16 ocular and visual symptoms related to the use of video display terminals. The frequency and intensity of use data are recoded to calculate the severity of each symptom, resulting in a total score. Total scores ≥ 6 indicate that the subject has computer vision syndrome.

## Data Availability

The datasets used and/or analyzed during the current study are available from the corresponding author on reasonable request.

## Abbreviations

CVS: Computer vision syndrome
VDTs: Video display terminals
CVS-Q^©^: Computer Vision Syndrome Questionnaire
