# Supplementary material for: The Chinese version of the Computer Vision Syndrome Questionnaire: translation and cross-cultural adaptation
Source: BMC Ophthalmol. 2023 Jul 3;23:298. doi: 10.1186/s12886-023-03031-y (PMC10318657; doi:10.1186/s12886-023-03031-y)
Supplement: Supplementary file 1 — Additional file 1. The CVS-Q CN©; 电脑视觉综合征调查问卷© – The Computer Vision Syndrome Questionnaire in Chinese (and its English version). It is a self-administered questionnaire in Chinese to evaluate the computer vision syndrome (or digital eye strain). This questionnaire assesses the frequency and intensity of 16 ocular and visual symptoms related to the use of video display terminals. The frequency and intensity of use data are recoded to calculate the severity of each symptom, resulting in a total score. Total scores ≥ 6 indicate that the subject has computer vision syndrome. [file 12886_2023_3031_MOESM1_ESM.pdf]

CVS-Q COMPUTER VISION SYNDROME QUESTIONNAIRE

To be completed by worker

Indicate whether you experience any of the following symptoms* during the time you use the computer at work. For each symptom, mark with an X:

a. First, the frecuency, that is, how often the symptoms occurs, considering that:
   NEVER = the symptom does not occur at all
   OCCASIONALLY = sporadic episodes or once a week
   OFTEN OR ALWAYS = 2 or 3 times a week or almost every day

b. Second, the intensity of the symptom:
   Remember: if you indicated NEVER for frequency, you should not mark anything for intensity.

* *If you regularly wear glasses or contact lenses when working with digital devices, you should answer by thinking about how you feel when you wear them*

	a. Frecuency			b. Intensity	
	NEVER	OCCASIONALLY	OFTEN OR ALWAYS	MODERATE	INTENSE
1. Burning					
2. Itching					
3. Feeling of a foreign body					
4. Tearing					
5. Excessive blinking					
6. Eye redness					
7. Eye pain					
8. Heavy eyelids					
9. Dryness					
10. Blurred vision					
11. Double vision					
12. Difficulty focusing for near vision					
13. Increased sensitivity to light					
14. Coloured halos around objects					
15. Feeling that sight is worsening					
16. Headache					

Intellectual property and conditions of use: https://www.biblioprointernational.org/en/
©All rights reserved

To be completed by investigator

Calculation of TOTAL SCORE, considering that:

- Frequency:
  - NEVER = 0
  - OCCASIONALLY = 1
  - OFTEN OR ALWAYS = 2
- Severity:
  - The result of Frequency x Intensity should be recorded as: 0 = 0; 1 or 2 = 1; 4 = 2
- Intensity:
  - MODERATE = 1
  - INTENSE = 2

	Frequency	Intensity	Frequency x Intensity	Severity
1. Burning				
2. Itching				
3. Feeling of a foreign body				
4. Tearing				
5. Excessive binking				
6. Eye redness				
7. Eye pain				
8. Heavy eyelids				
9. Dryness				
10. Blurred vision				
11. Double vision				
12. Difficulty focusing for near vision				
13. Increased sensitivity to light				
14. Coloured halos around objects				
15. Feeling that eyesight is worsening				
16. Headache				

TOTAL SCORE = $\sum_{i=1}^{16}$

If the total score is ≥ 6 points, the worker has Computer Vision Syndrome.

This is NOT the validated English version; it is only an English translation of the Chinese version.
The validated English version will be available in the BIBLIOPRO repository (https://www.biblioprointernational.org/en/)

Intellectual property and conditions of use: https://www.bibliointernational.org/en/
©All rights reserved
